# Microelectromechanical system‐based biosensor for label‐free detection of human cytomegalovirus

**DOI:** 10.1049/nbt2.12109

**Published:** 2022-12-20

**Authors:** Khalid E. Alzahrani, Abdulaziz K. Assaifan, Mahmoud Al‐Gawati, Abdullah M. Alswieleh, Hamad Albrithen, Abdullah Alodhayb

**Affiliations:** ^1^ Department of Physics and Astronomy College of Science King Saud University Riyadh Saudi Arabia; ^2^ Biological and Environmental Sensing Research Unit King Abdullah Institute for Nanotechnology King Saud University Riyadh Saudi Arabia; ^3^ Department of Biomedical Technology College of Applied Medical Sciences King Saud University Riyadh Saudi Arabia; ^4^ Department of Chemistry College of Science King Saud University Riyadh Saudi Arabia; ^5^ Research Chair for Tribology, Surface, and Interface Sciences Department of Physics and Astronomy College of Science King Saud University Riyadh Saudi Arabia

**Keywords:** bioMEMS, biomedical measurement, biosensors

## Abstract

The human cytomegalovirus (HCMV) is an asymptomatic common virus that is typically harmless, but in some cases, it can be life threatening. Thus, there is an urgent need to develop novel diagnostic methods and strengthen the efforts to combat this virus. A microcantilever‐based biosensor functionalised with the UL83‐antibody of HCMV (UL83‐HCMV antibody) has been developed to detect the UL83‐antigen of HCMV (UL83‐HCMV antigen) at different concentrations ranging from 0.3 to 300 ng/ml. The response of the biosensor to the presence of UL83‐HCMV antigen was measured through the shift in resonance frequency before and after antigen–antibody binding. The system shows a low detection limit of 84 pg/ml, which is comparable to traditional sensors, and a detection time of less than 15 min was achieved. The selectivity of the sensor was demonstrated using three different proteins with and without the UL83‐HCMV antigen. The biosensor shows high selectivity for the UL83‐HCMV antigen. Mass loading by the UL83‐HCMV antigen was roughly estimated with a sensitivity of ∼30 fg/Hz. This technique is crucial for the fabrication of portable and low‐cost biosensors that can be used in real‐time monitoring and enables early medical diagnosis.

## Graphical abstract

In this work, a microelectromechanical system‐based biosensor has been designed and successfully employed for the detection of human cytomegalovirus. The biosensor showed a low detection limit for HCM detection, making it a promising technology for the rapid and sensitive detection of HCMV.

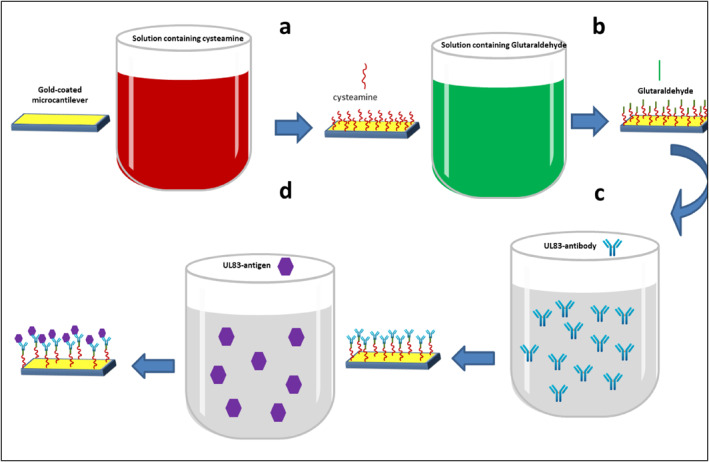

## 1 INTRODUCTION

Human cytomegalovirus (HCMV), also known as a human herpesvirus‐5 (HHV‐5), is a common cause of infections in approximately 60% of the population in developed countries and more than 90% in developing countries [1, 2, 3]. HCMV contains a double‐strand DNA genome wrapped with a protein complex called a capsid [4, 5]. Capsid and DNA are covered with a protein layer that is further covered by a lipid bilayer, known as envelop [6]. The interaction between the virus and its host cell is facilitated by various viral glycoproteins embedded within the envelop [4, 7]. HCMV infection is usually asymptomatic, but in some cases, it can be a serious and life‐threatening infection, for example, in immune‐compromised individuals, and may cause childhood disability due to in utero HCMV infection (5). Once the body is infected with HCMV, the elimination of the virus will be difficult, leading to chronic inflammation or the virus remaining latent in the host until further transmission to a new host [8, 9]. Thus, there is an urgent need to develop more novel diagnostic methods and strengthen the efforts to combat this virus.

Molecular techniques are the most widely used approaches for diagnosing the HCMV. Among the molecular techniques, PCR‐based methods are considered the gold standard [4, 10, 11, 12]. However, PCR‐based methods suffer from several drawbacks that hamper their use such as generating misleading results, the need for adequately trained personnel and complicated DNA extraction process [13]. Generally, the conventional methods are only appropriate for clinical laboratory‐based diagnosis. To overcome these limitations, huge efforts have been made for replacing the conventional techniques with new ones. Biosensors can have the potential to fulfil the aforementioned features. Biosensors are devices that are used to determine the presence of biological substances. Biosensing‐based methods are of importance for fabricating point‐of‐care devices that are likely to play a key role in the future for diagnosing and combating viral infections due to their capability of use at the site of need without complicated procedures associated with conventional methods [14, 15, 16, 17].

Biosensors can be classified based on their signal transductions. Electrochemical, optical and mechanical are the main types of transducers used in current biosensors. The electrochemical sensors are the most mature and widely used techniques for the detection of HCMV among biosensors. Authier et al. [18] developed a sensitive electrochemical DNA sensor by the hybridisation of the HCMV ssDNA with its complementary anchored to oligonucleotide‐functionalised Au nanoparticles. This DNA‐based biosensor could detect the 406‐base pair DNA sequence of HCMV at a concentration as low as 5 pM. Narang et al. [19] described an electrochemical DNA biosensor integrated with zinc–silver nanoblooms for the diagnosis of HHV‐5 virus, with a detection limit of 97 copies/ml. Pires et al. [20] proposed a method to detect glycoprotein B of HCMV (gB‐HCMV) in urine samples using magnetic particle‐based enzyme immunoassay (mpEIA), with a detection limit of 30 pg/ml. Huang et al. [21] reported an electrochemical amplification immunosensor for detecting the UL83‐antigen of cytomegalovirus using horseradish peroxidase attached to single‐walled carbon nanohorns decorated by Pt and Pd nanoparticles with a detection limit of 30 pg/ml.

Mechanical biosensors have gained attention owing to their low cost, sensitivity, ease of use and rapid response. The working principle of these types of sensors relies on transduction of bio‐recognition interactions into a mechanical signal. There are two main types of mechanical biosensors: cantilever‐based and piezoelectric. Microcantilever‐based biosensors are the most prominent techniques among the mechanical biosensors. In microcantilever‐based biosensors, there are two modes used for detecting: static mode (surface stress) and dynamic mode (mass sensing). In the static mode, the surface stress is generated by the binding of the target to the functional groups immobilised on the cantilever and this causes the cantilever to deflect. In the dynamic mode, the binding of molecules to the surface of the cantilever induces a change in the cantilever resonance frequency and the variation in the resonance frequency is used for the detection process. The mechanical sensors have been widely used to detect clinically important biological and chemical substances. A microcantilever modified with monoclonal antibodies (mAbs) or T8 has been used to detect envelope glycoprotein (gb120) of immunodeficiency virus type 1 (HIV‐1) with a detection limit of 8 μg/ml [22]. Cha et al. [23] reported the use of a silica nanoparticle‐enhanced dynamic microcantilever probe for the detection of hepatitis B Virus (HBV) DNA through DNA hybridisation. The biosensor can detect up to 23.1 fM of the HBV target DNA of 243 bp. Despite several studies on the diagnosis of HCMV using different biosensors, there is no or little reported literature on the use of mechanical biosensors [24, 25, 26, 27]. For example, quartz crystal microbalance (QCM) biosensors have been used for the diagnosis of distinctive antigens of HCMV. Yamamoto et al. [28] used QCM to detect the gB epitope of HCMV at a detection limit of 1 μg/ml.

In this work, a microcantilever‐based biosensor has been designed and successfully employed for HCMV detection. We report for the first time the detection of the UL83‐antigen of human cytomegalovirus (UL83‐HCMV antigen) using a microcantilever‐based biosensor. The surface of the microcantilever was modified with the UL83 antibody and used as receptors for the UL83 antigen. The biosensor showed a low detection limit for HCM detection.

## 2 MATERIAL AND METHODS

### 2.1 Materials

In this work, silicon microcantilever (OCTO500S, Micromotive, Germany) was used. Phosphate Buffer Saline (PBS) was purchased from Fisher Scientific, USA. Cysteamine and Glutaraldehyde (25% in water) were supplied by Sigma Aldrich, USA. UL83‐HCMV antibody was procured from Virusys‐corporation, USA. UL83‐HCMV antigen was purchased from Miltenyl Biotec Ltd., Surrey, UK. For selectivity experiments, bovine serum albumin (BSA) (Sigma Aldrich), low‐density lipoprotein (LDL)‐antigen (Sino Biological, China) and pepsin (SrL, India) were used.

### 2.2 Functionalisation of microcantilever sensor

**FIGURE 1:**
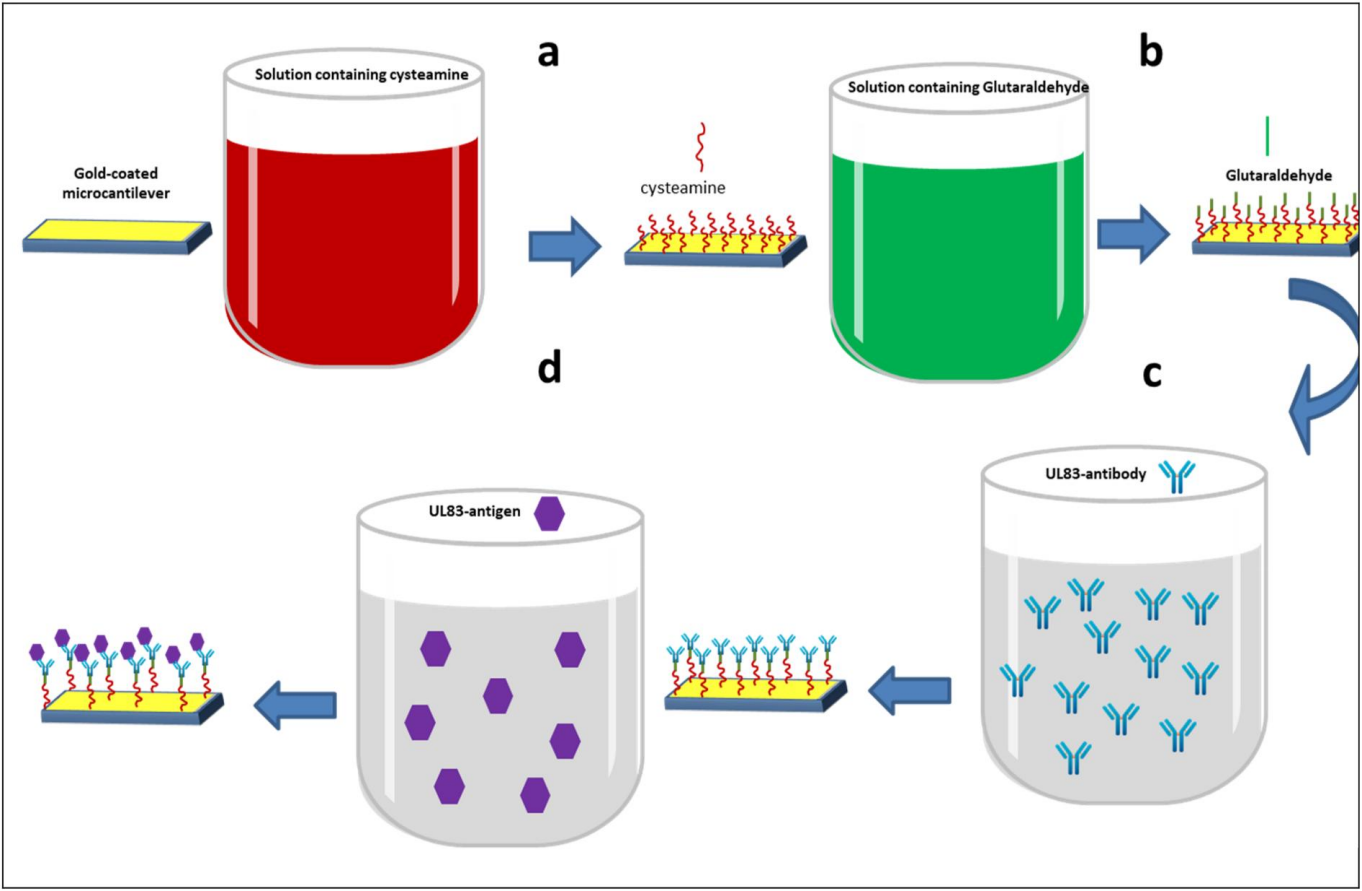
A schematic diagram showing the functionalisation steps of the microcantilever sensor.

Figure 1 shows a schematic diagram of functionalisation processes of the microcantilever sensor. Before coating with gold, the microcantilevers were cleaned with piranha solution to remove organic contaminants. Subsequently, the microcantilevers were coated with 40 nm gold film using vacuumed thermal evaporation system (Oerlikon, leybol vacuum, Germany); a 5‐nm chromium layer thermally deposited onto the microcantilever was used as an adhesive layer for the 40‐nm gold film. This process was carried out at rates of 2.25 Å/s and 0.1 Å/s for Au and Cr, respectively. The gold‐coated microcantilevers were incubated in an ethanolic solution of 1 mm cysteamine for 1 h at room temperature in darkness. The microcantilevers were rinsed using absolute ethanol. Subsequently, the microcantilevers were incubated in 2.5% glutaraldehyde in PBS for 1 h, followed by rinsing with deionised water. Then, the microcantilevers were incubated in 2.5% glutaraldehyde in PBS for 1 h, followed by rinsing with deionised water. Finally, the microcantilevers were incubated in 10 μg/ml of UL83‐HCMV antibody solution for 1 h, followed by rinsing with deionised water. As shown in Figure 1a, the terminal thiol groups of cysteamine bind to the surface of the gold‐coated microcantilever, while another terminal of the cysteamine containing amine groups covalently binds to the aldehyde groups of the glutaraldehyde, Figure 1b. The aldehyde groups serve as binding sites for the amine group of the UL83‐HCMV antibody, Figure 1c.

### 2.3 Measurements and sensing experiments

**FIGURE 2:**
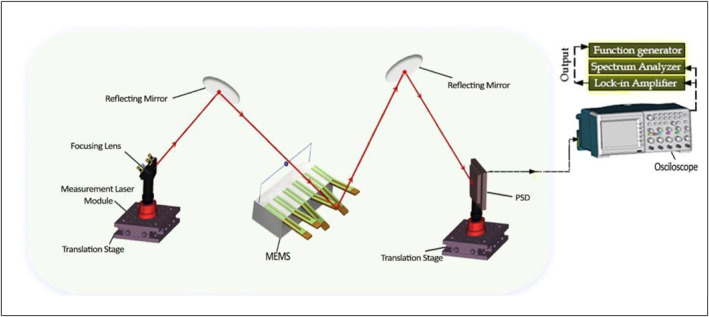
A schematic representation of the Picomeasure PM3 measurement system.

The frequency measurements of the microcantilevers were recorded by the Picomeasure PM3 system (FOURIEN, CANDA) [29, 30]. As demonstrated in Figure 2, a laser beam is focussed on the free end of the microcantilever. The reflective laser beam is detected by a position sensitive detector by which the mechanical deflection of the microcantilever is translated into an electrical signal, which is processed using connected electronics. The system can be operated in two different modes, namely, static and dynamic modes. The measurements have been performed in the dynamic mode, and the microcantilever was excited with a frequency range of 4–6 kHz. The microcantilever functionalised with the UL83‐HCMV antibody was immersed in UL83‐HCMV antigen solution for 15 min, Figure 1d. This process was repeated for various concentrations of UL83‐antigen ranging from 0.3 to 300 ng/ml.

To examine the selectivity of the biosensor, a mixture of different proteins has been used. The modified microcantilever was incubated for 15 min with a solution containing UL83‐HCMV antigen with a mixture of three proteins (BSA, LDL‐antigen, and pepsin), called a positive control sample. The experiment was repeated with the above mentioned mixture at the same concentration but without UL83‐HCMV antigen (as negative control sample).

## 3 RESULTS AND DISCUSSION

The surface properties of a microcantilever have a significant effect on its resonance frequency. The binding of target molecules on the microcantilever surface causes a change in its resonance frequency due to the surface stress induced by the molecules adsorption to the surface [31]. The microcantilever sensors were immersed stepwise in cysteamine, glutaraldehyde, and finally in a solution containing the UL83‐HCMV antibody. The cysteamine can be easily bound to the gold through the thiol groups and then its reaction with glutaraldehyde provides a linker for cross‐linking the UL83‐HCMV antibody to the biosensor. The attachment of the antibody to the cysteamine/glutaraldehyde layer forms a sensing layer for UL83‐HCMV antigen.

**FIGURE 3:**
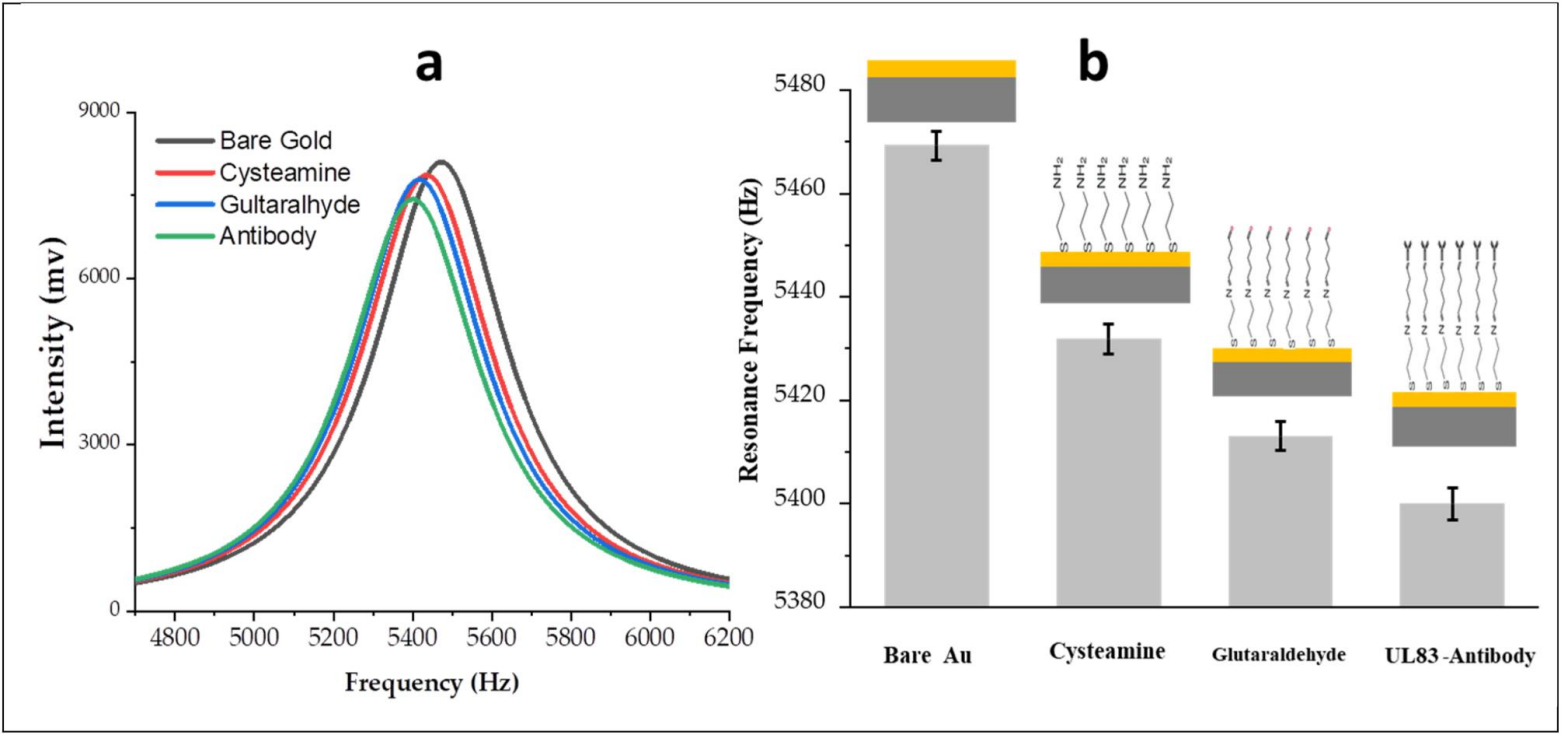
Change of resonance frequency of a gold‐coated microcantilever at different modification steps.

To assess the effectiveness of the immobilisation procedure of the sensing layer on the microcantilever, the resonance frequency of the microcantilever was recorded after each step of the surface modification. As depicted in Figure 3, chemisorption of cysteamine on the gold‐coated microcantilever causes a distinct shift of its resonance frequency by approximately 44 Hz. The resonance frequency was further decreased by approximately 18 Hz after the reaction of amine group with glutaraldehyde. The frequency measurements performed after incubation of the microcantilever‐based biosensors with a solution containing the UL83‐HCMV antibody show more resonance frequency change that can be attributed to the binding of the antibodies to the surface of biosensor. These results can be considered as a confirmation of the successful formation of the sensing layer on the surface of the microcantilever‐based biosensor.

**FIGURE 4:**
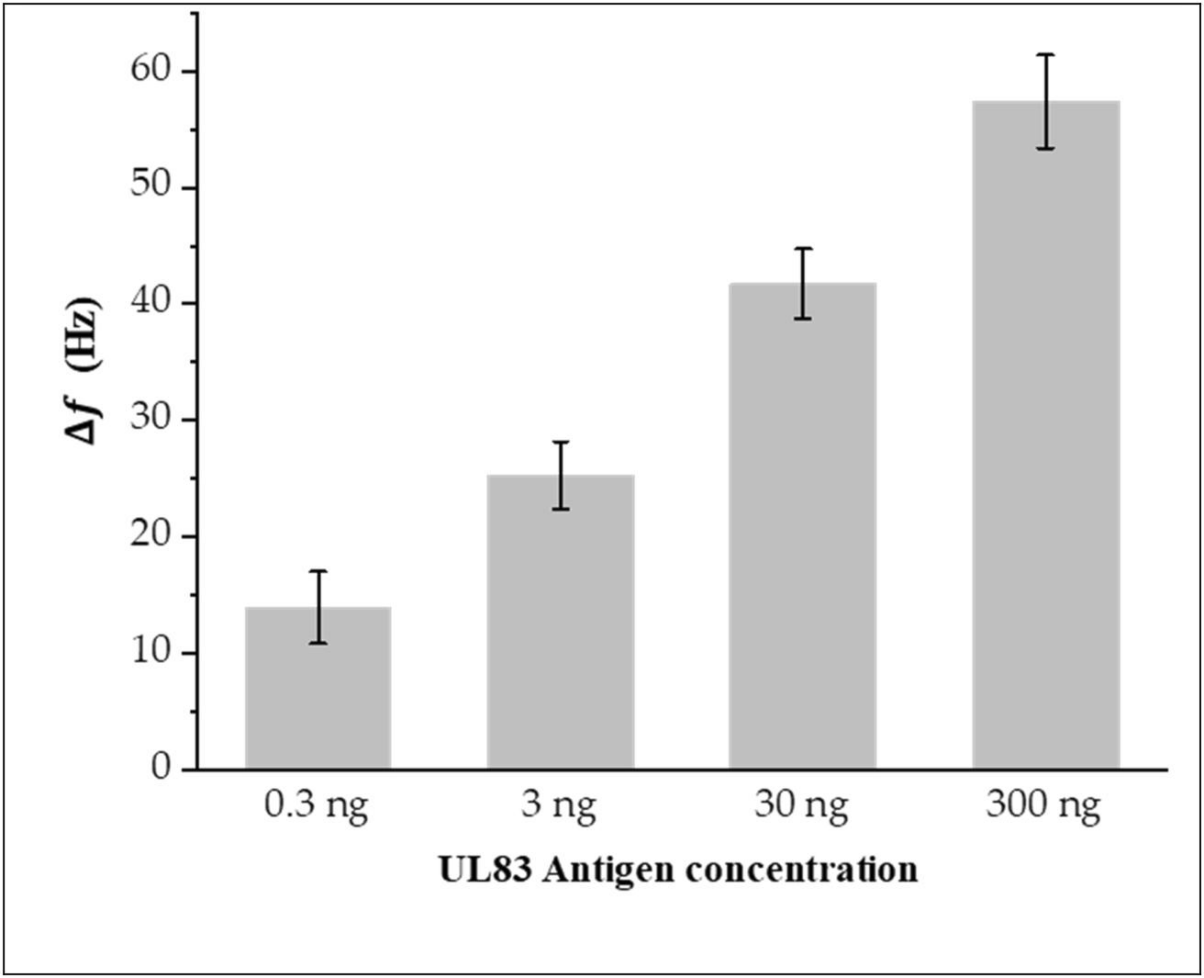
Changes in the resonance frequency of the microcantilever sensor at different concentrations of UL83‐HCMV antigen.

UL83‐HCMV antigen was selected as a target for detection because this antigen is the major target of the human immune system during the HCMV infection [32]. The microcantilever‐based biosensor functionalised with the UL83‐HCMV antibody was exposed to UL83‐HCMV antigen in increasing concentrations up to 300 ng/ml. The response of the microcantilever to the binding between antigens and antibodies can be monitored through the changes of the microcantilever resonance frequency, Figure 4. Here, the frequency response of the microcantilever decorated with the UL83‐HCMV antibody and only exposed to PBS solution could be considered as a reference (blank) for the subsequent measurements. The shift in the resonance frequency due to binding between UL83‐HCMV antigen and the UL83‐HCMV antibody ranges from ∼14 to ∼57 Hz for UL83‐HCMV antigen concentrations in the range between 0.3 and 300 ng/ml. The resonance frequency of the microcantilever decreased with increasing antigen concentration. It can be seen from Figure 3 that the resonance frequency drops by approximately 14 Hz after immersing the mcirocantilever in the solution containing 0.3 ng/ml of the antigens compared with a reference frequency, indicating that the attachment of the antigen to the sensor takes place. The detection time of the biosensor is about 15 min, making it one of the most rapid and simple biosensors in this field.

**FIGURE 5:**
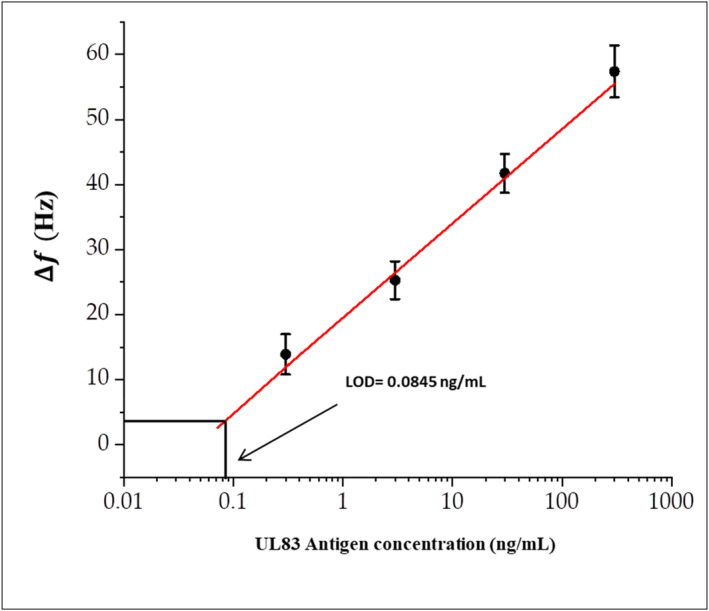
The calibration curve for the microcantilever biosensor.

Figure 5 shows the calibration curve of the biosensor derived from the resonance frequency shift of the microcantilever at different concentrations of UL83‐HCMV antigen. As can be extracted from the curve, the detection limit of the sensor (LOD) is estimated to be ∼84 pg/ml.

In comparison with other biosensors, our sensor still has high sensitivity and specificity. As mentioned above, UL83‐HCMV antigen has been detected using an electrochemical amplification immune‐sensor with a detection limit of 30 pg/ml. Another biosensor based on quantum dots and circular peptide‐DNA amplification demonstrated a lower detection limit of 0.33 fM for UL83‐HCMV antigen [33].

**FIGURE 6:**
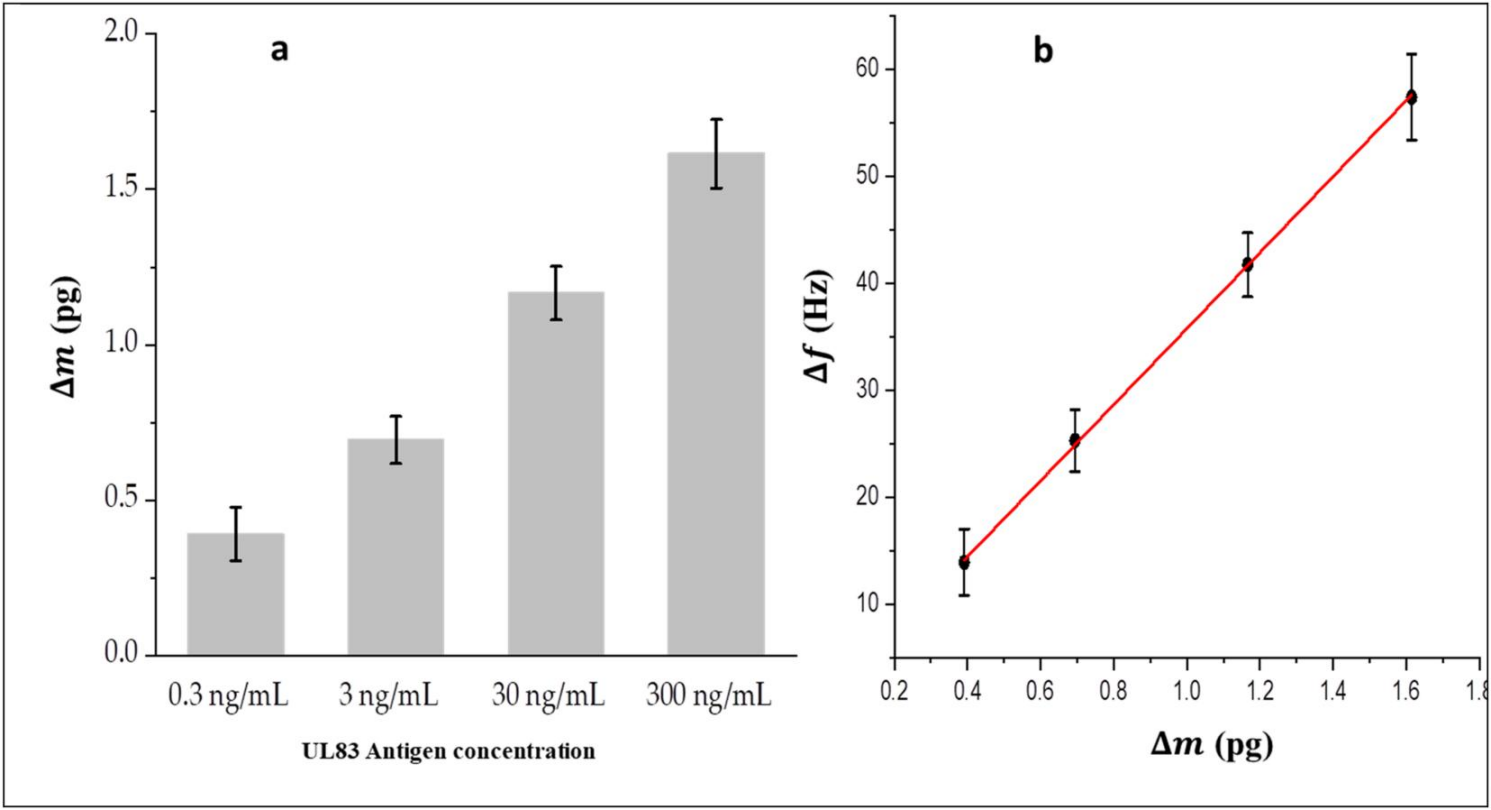
(a) Mass loading ∆m due to the antigen attachment to the microcantilever surface at different antigen concentrations and (b) ∆m as a function of microcantilever resonance frequency shift ∆f.

The resonance frequency of microcantilevers is directly affected by the binding of analytes to receptors anchored to the microcantilever through changing the overall mass of the microcantilever. Figure 6a shows concentration dependence of mass loading ∆m (the change in the microcantilever mass) due to antigen–antibody binding. Figure 6b illustrates a linear relation between ∆m and the frequency shift ∆f of the microcantilever because of UL83‐HCMV antigen attachment. ∆m can be roughly estimated using the following equation [34]

$$∆m=\frac{k}{{\left(2\pi \right)}^{2}n}\left(\frac{1}{{f_{1}}^{2}}-\frac{1}{{f_{0}}^{2}}\right)$$

Where k is the stiffness of the microcantilever (∼0.022 N/m), *n* is called a geometric factor (approximately 0.24 for a rectangle cantilever), and $f_{0}$, $f_{1}$ are the measured frequency before and after the attachment of antigens, respectively. However, the calculated mass does not provide accurate measurements but a rough estimation of the mass loading due to the binding of antigens.

The estimated change of mass due to the attachment of antigens is about 370 fg at a concentration of 300 pg/ml, and then increased to 1600 fg at a concentration of 300 ng/ml. Accordingly, the mass sensitivity that can be estimated from Figure 5b to be ∼30 fg/Hz.

**FIGURE 7:**
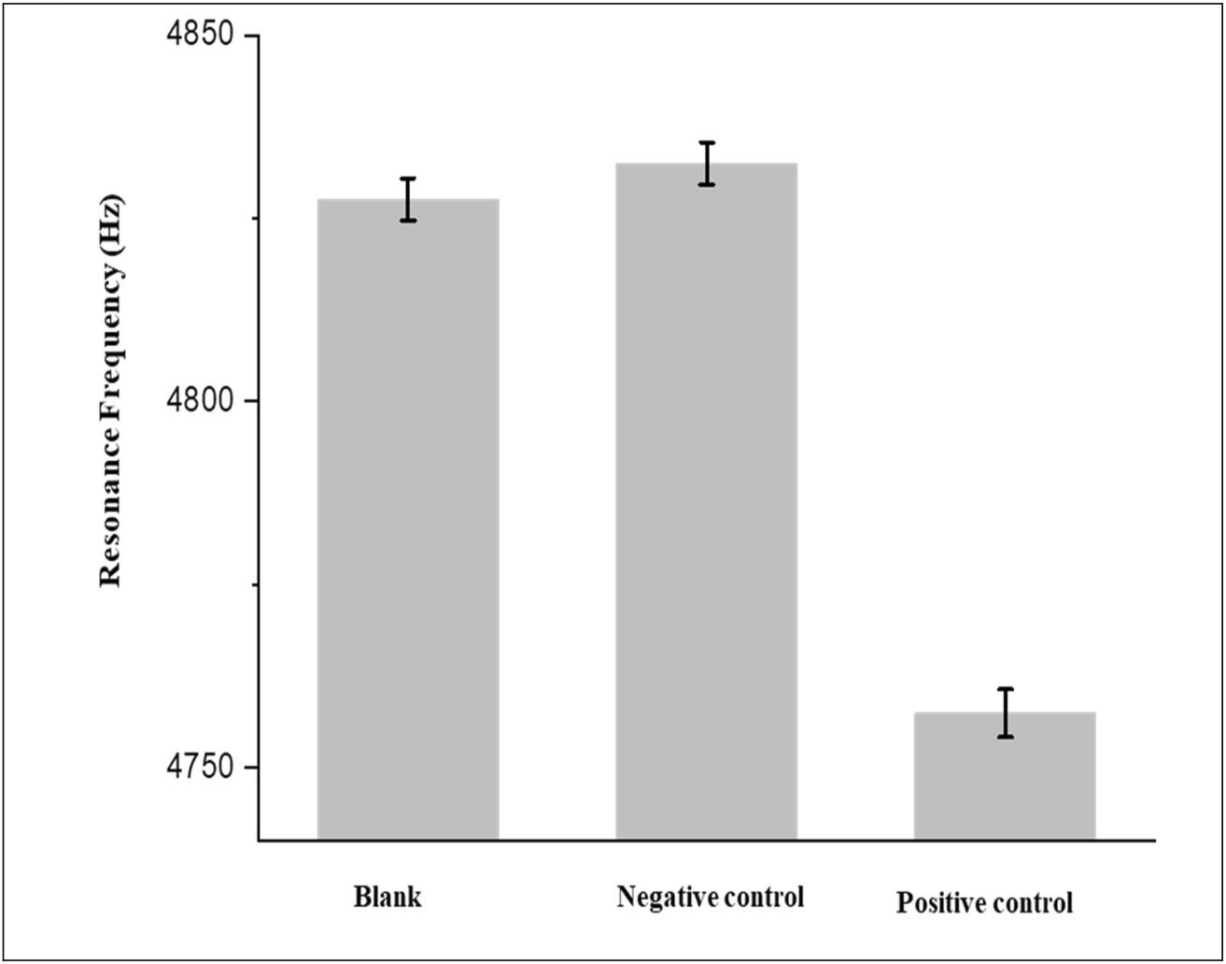
Selectivity test for the microcantilever‐based biosensor. The biosensor selectivity was tested with just Phosphate Buffer Saline (PBS) solution (blank), a mixture of three different proteins; bovine serum albumin (BSA), LDL‐antigen, pepsin without UL83‐HCMV antigen (negative control) and a mixture of proteins; BSA, LDL‐antigen, pepsin with UL83‐HCMV antigen (positive control).

To check the sensor’s selectivity to UL83‐HCMV antigen, a mixture of proteins containing BSA, LDL‐antigen, pepsin, and UL83‐HCMV antigen was employed (positive control sample). The same mixture of proteins but without UL83‐HCMV antigen was used (negative control sample). As illustrated in Figure 7, the microcantilever‐based biosensor shows no response after incubation in the negative control sample compared to that which was measured using PBS solution (blank). Figure 7 also shows a significant decrease in the resonance frequency of the microcantilever after incubation in the positive control sample. These results demonstrate that the biosensor is highly selective to the UL83‐HCMV antigen.

## 4 CONCLUSION

In this work, we demonstrate for the first time the label‐free detection of UL83‐HCMV antigen in low concentrations using a microcantilever‐based biosensor. Microcantilevers functionalised with the UL83‐HCMV antibody was exposed to different concentrations of UL83‐HCMV antigen ranging from 0.3 to 300 ng/ml. The results clearly indicate that the changes in the microcantilever resonance frequency are proportional to the target antigen concentrations. The estimated detection limit for UL83‐HCMV antigen is approximately 84 pg/ml. The specificity of the sensor was examined by using a mixture of three proteins. The results show that the biosensor is highly specific for UL83‐HCMV antigen. The detection technique reported here can be miniaturised and made portable. Hence, it allows on‐site detection of biological samples, such as saliva, and subsequently mass screening of diseases at low cost.

## AUTHOR CONTRIBUTIONS

**Khalid E. Alzahrani**: Supervision; Writing up; Conceptualisation. **Abdulaziz K. Assaifan**: Supervision; Funding; Conceptualisation. **Mahmoud Al‐Gawati**: Experiments. **Abdullah Alodhayb**: Supervision; Writing‐up. **Abdullah M. Alswieleh**: Data analysis. **Hamad Albrithen**: Supervision; Data analysis.

## CONFLICT OF INTEREST

The authors declare that they have no competing interests.

## Data Availability

The data that support the findings of this study are available from the corresponding author upon reasonable request.
